# Intravascular Imaging-Guided Percutaneous Coronary Intervention: Transforming Precision and Outcomes in Contemporary Practice

**DOI:** 10.3390/jcm14248883

**Published:** 2025-12-16

**Authors:** Malik Alqawasmi, James C. Blankenship

**Affiliations:** 1 Department of Internal Medicine, University of New Mexico, Albuquerque, NM 87131, USA; 2 Division of Cardiology, Department of Internal Medicine, University of New Mexico, Albuquerque, NM 87131, USA

**Keywords:** percutaneous coronary intervention, intravascular ultrasound, optical coherence tomography, stent optimization, coronary artery disease, image-guided revascularization, coronary stent failure prevention

## Abstract

Percutaneous coronary intervention (PCI) has evolved significantly over the past two decades, yet challenges in achieving optimal stent deployment and long-term outcomes persist, particularly in complex coronary anatomy. Intravascular imaging (IVI) modalities such as intravascular ultrasound (IVUS), optical coherence tomography (OCT), and near-infrared spectroscopy (NIRS) have transformed the precision of PCI by providing detailed cross-sectional visualization of vessel architecture, plaque morphology, and stent apposition. Compared to angiography-guided PCI, imaging-guided PCI enables more accurate lesion assessment, appropriate stent sizing, and detection of suboptimal results including under-expansion, malapposition, and edge dissections, factors strongly linked to restenosis and stent thrombosis. Large-scale randomized trials (e.g., ULTIMATE, ILUMIEN) and meta-analyses have demonstrated that imaging-guided PCI reduces major adverse cardiovascular events (MACE) and improves long-term stent patency, particularly in left main, bifurcation, and calcified lesions. Despite these benefits, adoption remains variable due to cost, procedural complexity, and training gaps. Emerging advances, including artificial intelligence-enhanced imaging, hybrid devices, and fusion of imaging with physiologic assessments, promise to integrate imaging more seamlessly into routine practice. This review summarizes current evidence, practical applications, and future directions of IVI-guided PCI, underscoring its growing role in contemporary interventional cardiology and its potential to personalize and optimize coronary revascularization strategies.

## 1. Introduction

**Figure 1:**
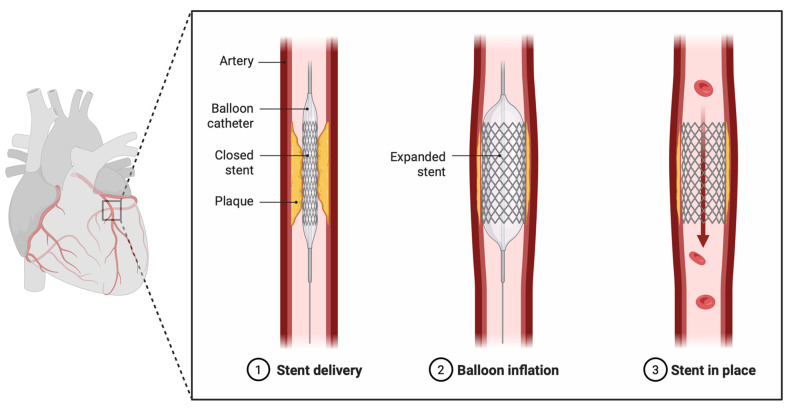
A visualization of percutaneous coronary intervention. Created in BioRender.com [3].

Percutaneous coronary intervention (PCI), as shown in Figure 1, has become a cornerstone of coronary revascularization, offering effective treatment for a broad spectrum of coronary artery disease (CAD), including stable angina and acute coronary syndromes [1]. Despite significant advances in stent technology, antithrombotic therapy, and procedural techniques, suboptimal PCI results remain a major contributor to adverse outcomes such as restenosis, stent thrombosis, and the need for repeat revascularization [2]. One of the key limitations of conventional angiography, the primary imaging tool during PCI, is its inability to provide detailed information about vessel dimensions, plaque characteristics, or the interaction between the stent and the vessel wall, especially given the two dimensional aspect of angiography [2].

The history of intravascular imaging (IVI) began in the late 1980s with the development of intravascular ultrasound (IVUS), which provided the first opportunity to visualize coronary arteries from within the vessel lumen [4]. IVUS enabled real-time, cross-sectional imaging that advanced the understanding of atherosclerotic plaque morphology and stent–vessel interactions beyond what was possible with angiography. In the early 2000s, optical coherence tomography (OCT) emerged, offering dramatically higher resolution through the use of near-infrared light [5]. OCT’s ability to visualize fine structures, such as thin-cap fibroatheromas, stent struts, and microdissections, represented a major leap forward in IVI [6]. Over time, both IVUS and OCT have evolved with advances in catheter design, image processing, and integration with interventional workflows, helping to refine PCI techniques and improve patient outcomes.

Alongside these structural imaging tools, near-infrared spectroscopy (NIRS) was developed to provide chemical composition analysis, specifically identifying lipid-core plaques associated with vulnerability to rupture [7]. NIRS is often integrated with IVUS in hybrid catheters to provide complementary structural and compositional data.

**Figure 2:**
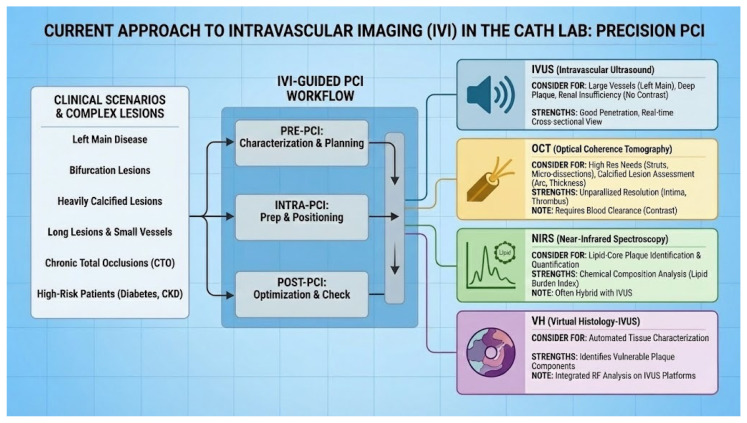
This illustration summarizes the current use of IVI to guide percutaneous coronary intervention (PCI), detailing its application across complex clinical scenarios and the key steps in the procedural workflow to achieve improved outcomes.

IVI modalities, including IVUS and OCT, as seen in Figure 2, have transformed interventional cardiology by providing high-resolution cross-sectional images of the coronary arteries [8]. These technologies enable precise lesion assessment, accurate stent sizing and positioning, and identification of mechanical issues that contribute to stent failure. A growing body of randomized trials and meta-analyses supports the use of IVI to improve procedural success and long-term outcomes, especially in complex cases involving left main disease [9], bifurcations [10], and heavily calcified lesions [11].

This review provides an updated perspective on IVI-guided PCI, covering the underlying rationale, supporting clinical evidence, practical considerations, current challenges, and future directions in precision coronary intervention.

## 2. Methods

This article is presented as a perspective, offering the authors’ views on the role of intravascular imaging in percutaneous coronary intervention. This perspective is informed by a selective review of the literature, based on searches of electronic databases (including PubMed, EMBASE, and the Cochrane Library) for articles published up to November 2025.

Key search terms included “percutaneous coronary intervention,” “PCI,” “image-guided PCI,” “intravascular imaging,” “IVI,” “intravascular ultrasound,” “IVUS,” “optical coherence tomography,” “OCT,” “stent optimization,” “major adverse cardiovascular events,” “MACE,” “target vessel failure,” and “stent thrombosis.”

The literature selection prioritized randomized controlled trials (RCTs), meta-analyses, observational studies, and key review articles addressing the comparison between imaging-guided and angiography-guided PCI. The focus was on publications reporting procedural outcomes, stent optimization, and long-term clinical endpoints. Articles not published in the English language or those not directly relevant to the clinical application of IVUS and OCT in PCI were excluded. The final body of literature was synthesized to inform the evidence and viewpoints presented in this article.

## 3. Technological Overview of Intravascular Imaging

IVI technologies have advanced the understanding and treatment of coronary artery disease by providing detailed, cross-sectional visualization of the vessel lumen and wall that cannot be obtained through angiography alone [12]. The two most widely used IVI modalities in contemporary practice are IVUS and OCT. Each modality, as shown in Table 1, offers distinct technical principles, strengths, and limitations that inform their application during PCI.

### 3.1. IVUS

IVUS employs high-frequency ultrasound waves, with newer generation catheters operating between 20 and 60 MHz. It uses a miniaturized piezoelectric transducer to generate real-time, 360° cross-sectional images of the target vessel [13]. The system differentiates tissue structures based on their echogenicity: echogenic materials like fibrous tissue and calcium produce bright, hyperechogenic signals, while echolucent lipid collections appear as dark, hypoechogenic signals. This allows for detailed characterization of plaque morphology, plaque burden, and vessel wall architecture. IVUS offers good tissue penetration, making it particularly valuable for assessing large vessels, such as the left main coronary artery [14], and for characterizing deep plaque morphology.

Modern IVUS catheters utilize two main engineering designs, solid-state (a circular phased array of transducers) and mechanical-state (a solitary rotating transducer), available in various crossing profiles and configurations. Image acquisition is performed via a manual or, preferably, an automated pullback. Automated pullback, which uses a “sled” to retract the catheter at a constant rate, is essential for accurate lesion length measurement. While traditional pullback speeds were 0.5–1.0 mm/s, newer iterations feature accelerated speeds up to 10 mm/s [2].

Recent technological progress has also improved imaging resolution. While historically around 70 µm, newer “high-definition” systems provide greater spatial resolution. Advanced platforms also integrate features like angiographic coregistration (overlaying the IVUS image on the angiogram) and radiofrequency (RF) analysis, such as Virtual Histology (VH-IVUS). This RF analysis, on platforms such as the Eagle Eye Gold catheter from Philips Volcano Corporation, provides automated tissue characterization to help identify vulnerable plaques, including thin-cap fibroatheromas, though its specificity can be limited by resolution [15].

### 3.2. OCT

OCT uses near-infrared light to produce high-resolution images with unparalleled detail of the intimal layer, stent struts, and thrombus [16]. OCT enables precise measurement of stent expansion, identification of malapposition, and detection of edge dissections or tissue prolapse [17]. However, its limited depth of penetration and the need for transient blood clearance during imaging can present procedural challenges, especially in cases of poor flow or hemodynamic instability [8].

OCT catheters emit near-infrared light from a rotating single optical fiber to generate real-time, high-resolution cross-sectional images. This technology provides the highest spatial resolution (axial 10–20 µm) of any available IVI modality, nearly 10 times greater than IVUS. This allows for unparalleled detail of the lumen-intima interface, stent struts, thrombus, and fine structures like micro-dissections [16,17].

This high resolution, however, comes at the expense of lower tissue penetration (1–2 mm). Furthermore, OCT requires a “bloodless” field of view, as light is attenuated by blood. This necessitates vessel clearance with contrast, which can be a limitation in certain lesion subsets, such as ostial lesions (due to blood admixture) or in patients where contrast volume is a primary concern [2,8].

Current OCT systems feature automated pullback to provide accurate lesion length and automated measurements of luminal dimensions. Advanced platforms also offer angiographic coregistration and are beginning to integrate artificial intelligence (AI)-based algorithms to aid in calcium detection and treatment planning. Hybrid IVUS-OCT systems, which aim to provide both deep penetration and high resolution from a single catheter, are also in development [18].

### 3.3. NIRS

NIRS is a unique intravascular imaging modality that provides functional, chemical data rather than structural images [2]. It operates on the principle that different substances, particularly lipids, absorb and scatter near-infrared light (wavelengths 800–2500 nm) in distinct ways. The system uses a scanning laser to analyze tissue composition, generating a color-coded “chemogram” that maps the distribution of lipids within the plaque [19].

NIRS is primarily used to identify and quantify lipid-core plaques, a key component of vulnerable plaque. Its main quantitative output is the Lipid Core Burden Index (LCBI) [19]. The finding of a large lipid-rich plaque (high LCBI) has been strongly associated with an increased risk of future adverse events from non-culprit lesions [20]. NIRS is almost exclusively used in a hybrid catheter combined with IVUS (NIRS-IVUS), which allows for simultaneous assessment of plaque composition (NIRS) and vessel structure (IVUS) during a single pullback.

Emerging technologies, including NIRS-IVUS hybrid catheters and artificial intelligence-assisted imaging platforms, aim to further enhance plaque characterization, automate image interpretation, and integrate physiologic and anatomic data to optimize PCI outcomes [7,21].

### 3.4. Rationale for Imaging-Guided PCI

Conventional invasive coronary angiography has long served as the foundation for guiding PCI. However, angiography is a two-dimensional luminogram that provides limited information about vessel geometry, plaque composition, and the interaction between stent and vessel wall [2]. This limitation contributes to risks of stent under-expansion, malapposition, geographical miss, and unrecognized edge dissections. These mechanical factors are well-established predictors of adverse outcomes, including restenosis, stent thrombosis, and target lesion failure [22,23].

IVI offers critical insights that complement and enhance angiographic guidance. By providing cross-sectional visualization of the vessel lumen and wall, IVUS and OCT enable accurate measurement of vessel dimensions, identification of appropriate landing zones, characterization of plaque morphology, and assessment of lesion severity beyond what is visible on angiography. These features are particularly valuable in complex lesions, such as those involving left main disease, bifurcations, long lesions, or heavily calcified plaques [9,10,11].

During PCI, imaging can guide lesion preparation, inform stent sizing, and help optimize stent deployment. Post-stent imaging enables detection and correction of mechanical complications, such as under-expansion or edge dissections, which might otherwise be missed and lead to adverse events. By addressing these procedural vulnerabilities, imaging-guided PCI has the potential to improve both short- and long-term outcomes, reduce the need for repeat interventions, and personalize revascularization strategies for patients with coronary artery disease [23].

Recent analyses reinforce the critical prognostic weight of these mechanical insights. A 2024 substudy of the OCTOBER trial [24] identified unintended stent deformation, a complication often angiographically invisible, in 9.3% of bifurcation cases and 18.5% of left main interventions. Crucially, while untreated deformation was associated with a 23.3% rate of major adverse cardiac events at two years, patients with corrected deformation experienced zero events. Similarly, the ILUMIEN IV trial [25] confirmed that minimum stent area (MSA) is the primary determinant of patency, with every 1 mm^2^ increase in MSA reducing the risk of target lesion failure by 24%. Furthermore, proximal edge dissections were identified as specific hazards, independently increasing adverse event risk by nearly two-fold. These findings underscore that “good enough” angiographic appearances are insufficient, and that achieving specific imaging-derived endpoints is non-negotiable for long-term safety.

### 3.5. Key Clinical Evidence

A growing body of clinical evidence supports the use of IVI to improve the safety and efficacy of PCI. Randomized controlled trials, observational studies, and meta-analyses have demonstrated that imaging-guided PCI reduces major adverse cardiovascular events (MACE), enhances stent deployment, and lowers the risk of restenosis and stent thrombosis, particularly in complex lesions [26,27,28,29,30].

IVUS has been extensively studied. The ULTIMATE trial [29] demonstrated that IVUS-guided drug-eluting stent (DES) implantation significantly reduced target vessel failure at three years compared to angiography-guided PCI. Similarly, the IVUS-XPL [28] trial in long coronary lesions showed that IVUS guidance reduced the composite of cardiac death, myocardial infarction, and target lesion revascularization. The ADAPT-DES study [31], a large registry, linked IVUS-guided PCI to reduced stent thrombosis rates and improved long-term outcomes.

OCT has been investigated in trials such as ILUMIEN III [32], which compared OCT, IVUS, and angiography guidance in complex PCI. The study found that OCT-guided stent sizing was non-inferior to IVUS for minimum stent area while providing superior resolution for detecting malapposition and edge dissections. The OPINION trial [33] and subsequent meta-analysis [34] demonstrated comparable outcomes between OCT- and IVUS-guided PCI.

Meta-analyses have consistently shown that imaging guidance reduces stent-related adverse events and the need for repeat revascularization [11,34]. These benefits are most pronounced in complex anatomies such as left main, bifurcation, and heavily calcified lesions. Despite these findings, real-world adoption of IVI remains variable, highlighting the need for continued education and guideline integration.

The clinical utility of intravascular imaging in complex PCI is robustly demonstrated by the Number Needed to Treat (NNT). Randomized data, such as findings from the RENOVATE-COMPLEX-PCI trial [6], indicate an NNT of approximately 22 to prevent one target vessel failure event over a median follow-up of 2.1 years. This therapeutic efficiency is even more pronounced in higher-risk anatomical subsets; meta-analyses suggest an NNT as low as 11 for unprotected left main interventions, compared to approximately 27 for bifurcation lesions. Consequently, for every ~22 complex procedures guided by imaging rather than angiography alone, one major adverse cardiac event, comprising cardiac death, target-vessel myocardial infarction, or clinically driven revascularization, is effectively prevented.

### 3.6. Imaging-Guided PCI Workflow

The integration of IVI into PCI requires a structured workflow that maximizes the benefits of these technologies at each procedural stage. A systematic approach to imaging-guided PCI enhances lesion assessment, guides optimal stent selection, and ensures high-quality procedural outcomes [35].

Pre-PCI imaging is critical for lesion characterization and procedural planning [36]. IVUS and OCT provide accurate measurements of reference vessel diameters, lesion length, and plaque burden. These measurements inform appropriate stent sizing and identify optimal landing zones free of significant disease. In addition, imaging can reveal features such as heavy calcification, lipid-rich plaques, or thrombus, which may influence the need for lesion preparation with atherectomy or intravascular lithotripsy.

Intra-PCI imaging assists in confirming the adequacy of lesion preparation, particularly in calcified lesions where balloon expansion may be suboptimal. It can also guide complex maneuvers, such as stent positioning in bifurcation lesions or left main interventions, where precision is critical to protect side branches and achieve complete coverage.

Post-stent imaging ensures that key mechanical endpoints are met [37]. These include achieving adequate stent expansion, typically defined by a minimum stent area appropriate for the vessel segment, confirming complete apposition of stent struts, and excluding complications such as edge dissection, tissue prolapse, or residual disease at stent margins. Timely detection of these issues allows for immediate correction, which is associated with improved long-term outcomes.

This stepwise use of imaging transforms PCI from a largely angiography-driven procedure into a precision-based intervention tailored to individual patient and lesion characteristics.

### 3.7. Special Scenarios

IVI plays an especially valuable role in complex and high-risk PCI scenarios where angiography alone may be insufficient to guide optimal treatment. These challenging settings highlight the strengths of both IVUS and OCT in achieving procedural precision and improving outcomes.

Left main coronary artery disease is one of the most important indications for imaging guidance. IVUS, with its ability to visualize larger vessels and deep structures, is considered the standard for left main PCI [9]. It enables accurate sizing, determination of plaque distribution, and confirmation of adequate stent expansion, which are critical in this large-caliber, high-flow vessel where adverse events can have serious consequences.

Bifurcation lesions benefit from imaging to guide stent strategy, optimize main vessel and side branch stent expansion, and ensure full coverage of the carina. Imaging can help determine whether provisional stenting is sufficient or whether a two-stent strategy is warranted [10].

Calcified lesions often require lesion preparation to facilitate full stent expansion. Imaging identifies calcium thickness, arc, and depth, enabling selection of appropriate adjunctive therapies such as rotational atherectomy, orbital atherectomy, or intravascular lithotripsy [11].

Chronic total occlusions (CTO) present a unique challenge. Imaging can assist in confirming true lumen position after wire crossing, guide stent sizing and placement, and ensure complete coverage of diseased segments [38].

Small vessel disease [39], diffuse long lesions [6], and high-risk populations [40], including patients with diabetes or chronic kidney disease, also benefit from IVI. In these cases, precise sizing and optimization are crucial to minimizing restenosis risk and maximizing long-term patency.

The ability of IVI to tailor PCI strategy to lesion complexity underscores its importance in contemporary interventional practice.

### 3.8. Barriers and Challenges

Despite robust evidence favoring intravascular imaging (IVI), global adoption remains suboptimal [41]. Bridging the gap between clinical guidelines and real-world practice requires moving beyond identifying problems to implementing systemic solutions regarding economics, education, and workflow.

#### 3.8.1. Economic Misalignment vs. Value-Based Care

High upfront costs for catheters and consoles create a disincentive in healthcare models prioritizing short-term cost containment over long-term outcomes [42]. Reimbursement often fails to cover the immediate expense of the device, leading to hospital-level restrictions [43].

To overcome this, healthcare systems must transition to value-based reimbursement models that account for the reduction in target vessel failure and repeat revascularization. Recent analyses demonstrate that IVI-guided PCI is cost-effective long-term by preventing costly readmissions. Administrators should advocate for “bundled” payments or specific Diagnosis-Related Group (DRG) modifiers that incentivize precision PCI, particularly for complex lesions where the economic benefit is highest.

In resource-limited settings where IVI is unavailable, optimized angiographic guidance remains the standard. Evidence from the GUIDE-DES trial [44] suggests that rigorous Quantitative Coronary Angiography (QCA) can achieve outcomes non-inferior to IVUS when strictly applied. In these scenarios, operators should maximize precision by utilizing orthogonal views to minimize foreshortening, employing digital QCA tools for objective sizing, and routinely performing high-pressure post-dilation to ensure adequate stent expansion, thereby mitigating the mechanical risks typically detected by imaging.

#### 3.8.2. The Expertise Gap and Educational Standardization

Image interpretation has a steep learning curve; variability in operator skill leads to underutilization or incorrect image assessment [42]. Currently, training is often carried out in an ad hoc manner rather than a mandatory component of interventional fellowships.

Standardization is required at the institutional and board-certification levels. IVI competency should become a mandatory milestone in interventional cardiology training programs. Furthermore, the integration of Artificial Intelligence (AI) can serve as a “digital mentor,” providing real-time, automated interpretation to reduce inter-operator variability and democratize expert-level assessment for less experienced operators.

#### 3.8.3. Operational Efficiency and Workflow Integration

Perceptions of prolonged procedure time and the complexity of catheter setup hinder routine use. Additionally, the need for contrast displacement in OCT requires contrast injection to clear blood from the imaging field [2], which poses risks for patients with renal dysfunction.

Hospitals must implement “always-on” imaging workflows where catheters are pre-connected and integrated into the sterile field, reducing setup friction. To address renal concerns, operators can adopt specific zero-contrast IVUS protocols for CKD patients, positioning IVUS as the standard of care in this demographic. Finally, the adoption of hybrid catheters (IVUS-OCT) could eliminate the need to switch devices, streamlining the decision-making process into a single pullback.

#### 3.8.4. Policy and Guideline Enforcement

Inconsistent integration of imaging into standardized hospital protocols results in selective rather than systematic use. Institutions should develop mandatory “checklists” for complex PCI (e.g., Left Main, CTO) that require imaging justification if not used. National guidelines must continue to elevate the class of recommendation for IVI, empowering quality improvement committees to track imaging utilization as a key performance metric for the cardiac catheterization lab.

## 4. Limitations of Evidence and Areas of Controversy

While the aggregate data favors IVI, it is essential to acknowledge specific technical limitations and “gray zones” where the clinical benefit is less absolute. The literature does not uniformly support the superiority of one modality over another, nor does it ignore the procedural risks associated with image acquisition.

### 4.1. Comparative Equivalence and the “Ceiling Effect”

Despite technological advancements, head-to-head comparisons between IVUS and OCT often yield neutral results rather than clear superiority. The ILUMIEN III trial [32] demonstrated that OCT-guided stent sizing was non-inferior, but not superior, to IVUS regarding minimum stent area. Similarly, the OPINION trial [45] found comparable clinical outcomes between the two modalities. This suggests a potential “ceiling effect” where the mechanical benefit of imaging guidance may plateau regardless of the specific higher-resolution features of OCT, particularly in hands of experienced operators.

This plateau extends to comparisons with optimized angiography. The 2024 GUIDE-DES trial [44] reported that Quantitative Coronary Angiography (QCA) was non-inferior to IVUS for 12-month target lesion failure. These results suggest that rigorous angiographic standardization can yield comparable outcomes to imaging guidance, further challenging the assumption that IVI is universally superior in all clinical contexts.

### 4.2. Technical “Blind Spots” and Interpretation Challenges

Each modality possesses inherent blind spots that complicate decision-making. VH-IVUS, while promising for tissue characterization, suffers from limited specificity due to resolution constraints [15]. Conversely, OCT’s high resolution comes at the cost of low tissue penetration (1–2 mm), making it unable to accurately assess residual plaque burden at stent edges or visualize deep vessel architecture in large arteries [8]. These limitations create a “gray zone” in lesion assessment where neither modality provides a complete physiological and anatomical picture independently.

### 4.3. Procedural Risks in Vulnerable Populations

The safety profile of IVI is not without caveats. A significant controversy surrounds the use of OCT in patients with renal insufficiency. Because OCT necessitates a “bloodless” field achieved via contrast injection, it poses a procedural risk for contrast-induced nephropathy. In clinical scenarios involving ostial lesions or hemodynamic instability, the requirement for blood clearance renders OCT technically challenging or contraindicated [46]. These factors highlight that while IVI improves precision, it introduces specific procedural variables that must be weighed against patient safety profiles.

## 5. Discussion

The body of evidence supporting IVI-PCI has grown substantially, transitioning IVI from an adjunctive tool to a foundational component of precision PCI. Large-scale trials consistently show that IVUS and OCT reduce MACE compared to angiography alone, particularly in complex scenarios. This benefit is driven by IVI’s ability to ensure optimal stent deployment by correcting under-expansion, malapposition, and edge dissections that are often angiographically silent yet serve as the primary nidus for stent failure. Despite this robust evidence, a significant implementation gap persists due to cost, reimbursement, and the operator learning curve associated with image interpretation. For IVI to become the standard of care, it must be not only clinically effective but also efficient and accessible.

The future of IVI-guided PCI will be defined by technologies that directly address these barriers, particularly through the deep integration of artificial intelligence (AI) and machine learning. AI is poised to help with the challenges of interpretation time and operator variability by providing real-time, automated decision support. Future platforms will likely move beyond simple measurements to offer an immediate interpretation of the pullback, identifying plaque morphology, flagging adverse features, and providing a “pass/fail” report on stent optimization. This will dramatically shorten the learning curve and make precision PCI faster and more reproducible.

Simultaneously, catheter technology is advancing toward hybrid systems that eliminate the current compromise between IVUS (penetration) and OCT (resolution). A single “one-pass” hybrid catheter could assess deep vessel structure and meticulously inspect the lumen-intima interface. The ultimate goal will be to fuse this anatomical data with functional assessment, integrating angiography-derived physiology (like QFR or vFFR) directly with the IVI pullback. This “function-and-structure” map will guide intervention with unprecedented precision, ensuring operators are optimizing not just the how of stenting, but also the where and why.

## 6. Recent Developments and Future Directions

The field of IVI-guided PCI continues to evolve rapidly, with ongoing innovations aimed at enhancing precision, simplifying use, and broadening accessibility. Several promising developments are poised to address current limitations and further integrate imaging into routine PCI practice.

AI and machine learning are being increasingly studied in IVI. AI algorithms can assist in the automated detection of key procedural targets, such as stent under-expansion, malapposition, edge dissections, and residual plaque burden. Real-time AI analysis may help standardize interpretation, reduce inter-operator variability, and support decision-making during PCI [47].

Hybrid imaging technologies, including NIRS-IVUS and ongoing efforts to combine OCT and IVUS capabilities, aim to provide comprehensive anatomic and compositional plaque assessment through a single catheter. These tools promise to streamline workflows and improve lesion characterization.

Integration of imaging and physiology represents another frontier. Future platforms may combine imaging with wire-free or angiography-derived physiology (such as QFR or vFFR) to provide both anatomic and functional assessment in a single procedure, reducing equipment needs and procedure time [48].

Robotic and remote PCI systems may incorporate IVI to enable precise, image-guided interventions at a distance, potentially expanding access to advanced PCI techniques in underserved areas [49]. Greater standardization of imaging criteria and widespread inclusion of imaging in PCI guidelines will be essential to promoting consistent, evidence-based use and improving patient outcomes globally.

Finally, two pivotal randomized trials currently awaiting results are expected to provide definitive evidence for specific high-risk subsets and economic viability. The OPTIMAL trial (ClinicalTrials.gov ID: NCT04111770) is evaluating the superiority of IVUS guidance versus qualitative angiography specifically in unprotected left main coronary artery disease, aiming to establish a definitive standard for this critical anatomical subset. Concurrently, the IMPROVE trial [50] (ClinicalTrials.gov ID: NCT04221815) is assessing the impact of IVUS on clinical outcomes and cost-effectiveness in a broad population of complex lesions. Results from these studies are expected to address remaining gaps regarding economic impact and potentially elevate guideline recommendations to the highest level.

## 7. Conclusions

IVI has emerged as a new tool in PCI, enabling a shift from angiography-guided to precision-guided coronary revascularization. By providing detailed visualization of vessel architecture, plaque characteristics, and stent–vessel interactions, IVUS and OCT enhance lesion assessment, stent selection, and optimization of procedural outcomes. Robust evidence from randomized trials and meta-analyses supports the role of imaging-guided PCI in reducing adverse events, particularly in complex lesions involving the left main artery, bifurcations, long or calcified segments, and in high-risk patient populations.

Despite these advantages, barriers such as cost, operator expertise, and variability in clinical practice have limited universal adoption. Future innovations, including artificial intelligence, hybrid imaging platforms, and the integration of imaging with physiological assessment, promise to further enhance the value and usability of IVI. A greater focus on education, standardized protocols, and supportive health policy will be essential to realizing the full potential of imaging-guided PCI in improving patient outcomes.

As PCI continues to advance, IVI will play an increasingly central role in delivering personalized, high-quality coronary intervention that meets the growing demands of contemporary cardiovascular care.

## Figures and Tables

**Table 1:** OCT: optical coherence tomography; IVUS: intravascular ultrasound; NIRS: near-infrared spectroscopy.

Characteristic	OCT ^A^	IVUS ^B^	NIRS ^C^
Energy Source	Near-infrared light	Ultrasound (20–60 MHz)	Near-infrared light
Wavelength (µm)	1.3	35–80	800–2500
Resolution (µm)	15–20 (axial); 20–40 (lateral)	100–200 (axial); 200–300 (lateral)	Low (not intended for morphological resolution)
Frame Rate (Frames/S)	15–20	30	N/A
Pull-Back Rate (mm/S)	1–3	0.5–1	0.5–1
Maximum Scan Diameter (mm)	7	15	15
Tissue Penetration (mm)	1–2.5	10	1–1.5
Blood Clearing	Required	Not required	Not required
Advantages	Can measure and characterize thickness of calcium	More favorable imaging in larger vascular structures (e.g., left main)	Detects lipid-core plaques; quantifies lipid burden (Lipid Core Burden Index)
Disadvantages	Cannot accurately assess residual plaque burden at stent edges	Inferior detection of stent malapposition and edge dissections	Cannot visualize lumen or stent apposition; low spatial resolution

^A^ Based on specifications of the LightLab M2/M3 time-domain OCT imaging system. ^B^ Based on specifications of current generations of Volcano, Boston Scientific, and Terumo IVUS systems. ^C^ Based on specifications of Infraredx, which is a hybrid NIRS-IVUS catheter system.

## Data Availability

Data sharing is not applicable to this article as no datasets were generated or analyzed during the current outline of advances in our field of expertise.
